# Are There Striking Differences in Outpatient Use of Antibiotics Between South Backa District, Serbia, and Some Scandinavian Countries?

**DOI:** 10.3389/fpubh.2018.00091

**Published:** 2018-03-29

**Authors:** Olga Horvat, Vesna Mijatović, Boris Milijasević, Ana Tomas, Milica Paut Kusturica, Zdenko Tomić, Ana Sabo

**Affiliations:** Department of Pharmacology, Toxicology and Clinical Pharmacology, Faculty of Medicine, University of Novi Sad, Novi Sad, Serbia

**Keywords:** antibiotics, outpatients, serbia, defined daily dose, pharmacoepidemiology

## Abstract

There is little published information about antibiotic utilization in outpatients in Serbia. The objective of this study was to determine the amount and structure of outpatient antibiotic use in South Backa District (SBD) in Serbia, to assess prescibing quality of antibiotics and to compare with results from Scandinavian countries. Data on the antibiotic use were collected from all private and state-owned pharmacies from January through March 2008 in SBD. Results were expressed as the number of defined daily doses/1,000 inhabitants/day. The drug utilization 90% method was also used. Penicillins were the most frequently used antibiotic subgroup in SBD (35.20%), followed by cephalosporins (19.16%) and macrolides (13.18%). Thirteen drugs accounted for 90% of total antibiotics consumption (DU90% segment). The average cost/DDD within the DU90% segment was 0.95 euros, whereas the average cost/DDD beyond the DU90% segment was 1.89 euros, indicating that less expensive antibiotics were more frequently used. High use of ampicillin, third-generation cefalosporins, co-trimoxazole, and gentamicin, will aggravate the alarming problem of resistance in Serbia. Differences in the amount and structure of antibiotic consumption between SBD and Scandinavian countries indicate the need of updated national guidelines for rational antimicrobial drug use in Serbia.

## Introduction

The current worldwide increase in antimicrobial resistance is multifactorial, but the leading cause is the high consumtion of antibiotics. Outpatient use of antibiotics accounts for about 80–90% of antibiotic sales worldwide (1). Thorough surveillance of outpatient antibiotic use is one of the strategies to manage and control innapropriate utilization of antibiotics. For that purpose, the European Surveillance of Antimicrobial Consumption (ESAC) project established an extensive database of outpatient antibacterial consumption in Europe (2, 3).

The Republic of Serbia is a southern European country undergoing a socio-economical transition. After Yugoslavia split, Serbia became an independent state in 2006. Serbia, together with Federation of Bosnia and Herzegovina, the former Yugoslav Republic of Macedonia and Montenegro, is one of the few countries not participating in the ESAC project.

Some endeavors have been made to monitor antibiotic use in Serbia. After Yugoslavia disintegrated, Serbia, started with implementation of educational (i.e., Continuing Medical Education) and administrative measures (related to promotion of rationalization of antibiotic use through restriction of available antibiotics funded by the Republic Fund of Health Insurance and introduction of capitation), in order to prevent overuse of antibiotics. However, the situation is still far from ideal because of several reasons. First, there is a need for updated national guidelines on antimicrobial use in the outpatient primary care. Second, private pharmacies are not completely implemented in the Health Insurance Institution and they are not so strictly controlled by the state. Finally, the system of health professionals’ continuous education in Serbia has only recently been implemented. Besides, there is an official statics on drug utilization of the Agency for Drugs and Medical Devices of the Republic of Serbia. However, the agency obtains data from actual drug sales from manufacturers or their representatives and does not report separately inpatient and outpatient consumption of antibiotics.

Because the information about antibiotic utilization in Serbia is scanty and there are rare publications on the topic and the ones we have are incomplete (4–6), especially about outpatient use, the aims of the present study were to determine the amount and structure of outpatient antibiotics use issued in all state-owned and private pharmacies in the South Backa District (SBD), to estimate prescribing quality of antibiotics (drug utilization 90% method), as well as to compare results of this study with those in Denmark and Finland (the countries with well-developed pharmacotherapeutic practices) for the same year.

## Materials and Methods

The investigation was carried out in SBD with over a 3-month period in 2008. SBD is one of 29 Serbian districts. It is situated in the northern part of the country with 605,720 inhabitants (according to the 2008 cenzus), which correspond to 8.2% of the total Serbian population and is representative for the whole Serbian population, in terms of demographics.

The data on the number of packages, size of packages, and retail price of antibiotics [anatomical therapeutic chemical (ATC) group J01] from 1 January to 31 March 2008 were obtained from all state-owned and private pharmacies in SBD. The number of defined daily doses per 1,000 inhabitants per day (DDDs/TID) was calculated using ATC/DDD methodology valid in 2008 (7). The proportion of parenteral use of the total outpatient use was assessed. Parenteral use was expressed as a percentage of the total outpatient use in DDDs/TID.

Drug utilization 90% methodology was also used. This ranks drugs by volume of DDDs and determines how many and which drugs account for 90% of total consumption. The principle is to focus on the drugs that account for 90% of the prescribed volume and the adherence to guidelines in this DU90% segment (8). The price per DDD for each antibiotic in the DU90% segment, mean total price per DDD for all antibiotics within and beyond the DU90% segment, and the mean price per DDD for all antibiotics dispensed in SBD over a 3-month period were also calculated.

Data on the antibiotic consumption in Denmark and Finland for the same year as in SBD were taken from the annual reports that are regularly issued in electronic format, and they represent outpatient consumption (9, 10).

## Results

Utilization of antibiotics for systemic use (ATC group J01) in SBD, Denmark, and Finland is presented in Table 1. The total outpatient utilization of group J01 antibiotics in the SBD (26.93 DDDs/TID) was higher than in Denmark (16.2 DDDs/TID) and in Finland (18.13 DDDs/TID).

**Table 1:** Total outpatient use of antibacterials for systemic use (J01) in South Backa District (SBD), Denmark, and Finland in 2008, expressed in defined daily doses per 1,000 inhabitants per day (DDDs/TID) and percentages.

ATC code	Name of therapeutic subgroup	SBD		Denmark		Finland	
		DDDs/TID	%	DDDs/TID	%	DDDs/TID	%
J01C	Penicillins	9.48	35.20	10	61.73	6.11	33.71
J01D	Cephalosporins	5.16	19.16	0	0	2.31	12.47
J01A	Tetracyclines	3.17	11.17	1.7	10.49	4.02	22.17
J01F	Macrolides, lincosamides	3.55	13.18	2.4	14.82	1.32	7.28
J01M	Quinolones	2.15	7.98	0.5	3.08	0.86	4.74
J01E	Sulfonamides and trimethoprim	1.86	6.91	0.8	4.93	1.44	7.94
	Other J01 Classes	1.56	5.71	0.8	4.63	2.07	11.72

J01	Total	26.93	100.0	16.2	100.0	18.13	100.0

*ATC, anatomical therapeutic chemical*.

Penicillins were the most frequently used antibiotic subgroup in all tree investigated countries. In SBD and Finland it accounted for approximatelly 35% of overall outpatient consumption, while in Denmark the use was almost twice as high (61.73%).

The main difference was observed in consumption of cephalosporins: in SBD and Finland the percentage of cephalosporins was similar (19.16 and 12.47%, respectively), while there was no consumption of cephalosporins at all in Denmark.

On the other hand, the percentage of tetracyclines consumption in Finland (22.17%) was approximately two-hold of that in Denmark (10.49%) and SBD (11.17%), but the percentage of macrolides consumption was approximately twice lower in Finland (7.28%) than in Denmark (14.82%) and SBD (13.18%).

The quinolones consumption was approximately two times as high in SBD than in Scandinavian countries and the consumption of sulfonamides and trimethoprim was lower in Denmark compared to SBD and Finland.

Distribution of drug utilization within the therapeutic subgroup of penicillins (J01C) expressed in DDDs/TID SBD is shown in Table 2.

**Table 2:** Utilization of antibiotics within penicillins subgroup (J01C) in South Backa District (SBD), Denmark, and Finland in 2008.

ATC code	Drug name	SBD		Denmark		Finland	
		Defined daily doses per 1,000 inhabitants per day (DDDs/TID)	Share (%)	DDDs/TID	Share (%)	DDDs/TID	Share (%)
J01CA01	Ampicillin^a^	1.12	11.8				
J01CA02	Pivampicillin			0.5	5		
J01CA04	Amoxicillin	5.71	60.2	1.3	13	2.65	43.37
J01CA08	Pivmecillinam			1.5	15	0.67	10.97
JO1CE02	Phenoxymethylpenicillin^a^	1.34	14.1	5.3	53	1.59	26.02
J01CE30	Procaine benzyl penicillin	0.17	1.8				
J01CF01	Dicloxacillin			1.1	11	0.02	0.33
J01CF02	Cloxacillin					0.01	0.16
J01CR02	Amoxicillin+clavulanic acid^a^	1.15	12.1	0.3	3	1.17	19.15

TOTAL J01C		9.48	100.0	10	100.0	6.11	100.0

*ATC, anatomical therapeutic chemical*.

*^a^Drugs fully reimbursed by the Republic Fund for Health Insurance of Serbia in 2008*.

The most frequently used penicillin in SBD and in Finland was amoxicillin (60.2 and 43.37%), while in Denmark it occupied the third place with 13%. In Denmark, phenoxymethylpenicillin was the most used antibiotic with 53%, while in SBD and Finland it occupied the second place with 14.1 and 26.02%.

Amoxicillin+clavulanic acid was the third most commonly used penicillin antibiotics in SBD and Finland (12.1 and 19.15%) while in Denmark in was on the last place within this subgroup with only 3%.

While in SBD, ampicillin in the form of capsules was still used and with 11.8% occupied forth place, in Scandinavian countries only a prodrugs of ampicillin, with greater lipophilicity and better oral bioavailability compared to that of ampicillin were used.

Penicillins for parenteral use were recorded in outpatients only in SBD. On the other hand, beta lactamase-resistant penicillins were used only in Denmark and Finland. The only representative on the market in Serbia, cloxacillin, was not reimbursed by the Republic fund for health insurance.

In cephalosporine subgroup (J01DA), the first-generation cephalosporins showed a dominant utilization in SBD and in Finland (Table 3), represented mainly with cephalexin (3.57 and 2.26 DDDs/TID, respectively). Out of the second-generation cefalosporins, cefaclor was most commonly used, while in Finland it was cefuroxime. Cefixime, the third-generation cefalosporine for oral use, was located on the third place in SBD; while in Scandinavian countries, the third-generation cefalosporins were not used at all.

**Table 3:** Utilization of cephalosporins (J01C) in South Backa District (SBD), Denmark, and Finland in 2008.

ATC code	Drug name	SBD		Denmark		Finland	
		Defined daily doses per 1,000 inhabitants per day (DDDs/TID)	Share (%)	DDDs/TID	Share (%)	DDDs/TID	Share (%)
J01DB01	Cefalexin^a^	3.57	69.00	–		2.16	93.51
J01DB05	Cefadroxil^a^	0.08	1.61	–		0.06	2.60
J01DC02	Cefuroxime	0.08	1.51	–		0.06	2.60
J01DC04	Cefaclor	0.13	2.60	–		0.03	1.30
JO1DC10	Cefprozil^a^	0.06	1.16	–			
J01DD04	Ceftriaxone	0.12	2.36	–			
J01DD08	Cefixime^a^	1.08	20.92	–			
J01DD14	Ceftibuten	0.04	0.78	–			

TOTAL J01D		5.17	100.0	–		2.31	100.0

*ATC, anatomical therapeutic chemical*.

*^a^Drugs fully reimbursed by the Republic Fund for Health Insurance of Serbia in 2008*.

While in SBD and Finland the utilization of doxycyclin was especially pronounced, in Denmark tetracycline was located on the first place (Table 4).

**Table 4:** Utilization of antibiotics within tetracyclines subgroup (J01A) in South Backa District (SBD), Denmark and Finland in 2008.

ATC code	Drug name	SBD		Denmark		Finland	
		DDDs/TID	Share (%)	DDDs/TID	Share (%)	DDDs/TID	Share (%)
**J01AA02**	Doxycycline^a^	3.09	97.47	0.6	35.3	2.39	59.45
J01AA04	Limecycline			0.3	17.6	0.67	16.67
J01AA06	Oxyitetracycline			0.1	5.8		
J01AA07	Tetracycline	0.08	2.53	0.7	41.7	0.96	23.88

TOTAL J01A		3.17	100.0	1.7	100.0	4.02	100.0

*ATC, anatomical therapeutic chemical*.

*^a^Drugs fully reimbursed by the Republic Fund for Health Insurance of Serbia in 2008*.

The utilization of long acting-macrolides (azithromycin) was dominant in SBD and Finland (Table 5). On the other hand, the intermediate acting macrolides (mainly roxithromycin) was predominantly used in Denmark.

**Table 5:** Utilization of antibiotics within macrolides and lincosamides subgroup (J01F) in South Backa District (SBD), Denmark and Finland in 2008.

ATC code	Drug name	SBD		Denmark		Finland	
		Defined daily doses per 1,000 inhabitants per day (DDDs/TID)	Share (%)	DDDs/TID	Share (%)	DDDs/TID	Share (%)
J01FA01	Erithromycin^a^	0.36	10.1	0.7	29.2	0.08	6.06
J01FA06	Roxithromycin	0.49	13.8	0.9	37.5	0.26	19.70
J01FA09	Clarithromycin^a^	0.78	22	0.3	12.5	0.36	27.27
J01FA10	Azithromycin^a^	1.72	48.5	0.5	20.8	0.57	43.18
J01FA15	Telithromycin					0.05	3.79
J01FF01	Clindamycin	0.20	5.6				

TOTAL J01F		3.55	100.0	2.4	100.0	1.32	100.0

*ATC, anatomical therapeutic chemical*.

*^a^Drugs fully reimbursed by the Republic Fund for Health Insurance of Serbia in 2008*.

Among quinolones, ciprofloxacine was the leading drug in all three investigated countries (Table 6). The use of pipemidic acid was recorded only in SBD.

**Table 6:** Utilization of antibiotics within quinolones subgroup (J01M) in South Backa District (SBD), Denmark, and Finland in 2008.

ATC code	Drug name	SBD		Denmark		Finland	
		Defined daily doses per 1,000 Inhabitants per day (DDDs/TID)	Share (%)	DDDs/TID	Share (%)	DDDs/TID	Share (%)
J01MA01	Ofloxacin^a^	0.07	3.43			0.05	5.81
J01MA02	Ciprofloxacin^a^	1.39	64.44	0.5	100	0.46	53.49
J01MA06	Norfloxacin^a^	0.11	5.28			0.12	13.95
J01MA12	Levofloxacin					0.16	18.60
J01MA14	Moxifloxacin					0.07	8.14
J01MB04	Pipemidic acid^a^	0.58	26.85				

TOTAL J01M		2.16	100.0	0.5	100.0	0.86	100.0

*ATC, anatomical therapeutic chemical*.

*^a^Drugs fully reimbursed by the Republic Fund for Health Insurance of Serbia in 2008*.

The J01E subgroup was represented only by co-trimoxazole (the only member on market, 1.86 DDDs/TID) in SBD, while in Denmark and Finland the utilization of thrimetoprim was pronounced (Table 7).

**Table 7:** Utilization of antibiotics within sulfonamides and trimethoprim subgroup (J01A) in South Backa District (SBD), Denmark, and Finland in 2008.

ATC code	Drug name	SBD		Denmark		Finland	
		Defined daily doses per 1,000 Inhabitants per day (DDDs/TID)	Share (%)	DDDs/TID	Share (%)	DDDs/TID	Share (%)
J01EE01	Co-trimoxazole^a^	1.86	100			0.04	2.78
J01EE02	Sulfadiazine and trimethoprim					0.33	22.92
J01EA01	Trimethoprim			0.5	62.5	1.07	74.31
J01EB02	Sulfamethizol			0.3	37.5		

TOTAL J01E		1.86	100.0	0.8	100.0	1.44	100.0

*ATC, anatomical therapeutic chemical*.

*^a^Drugs fully reimbursed by the Republic Fund for Health Insurance of Serbia in 2008*.

**Figure 1:**
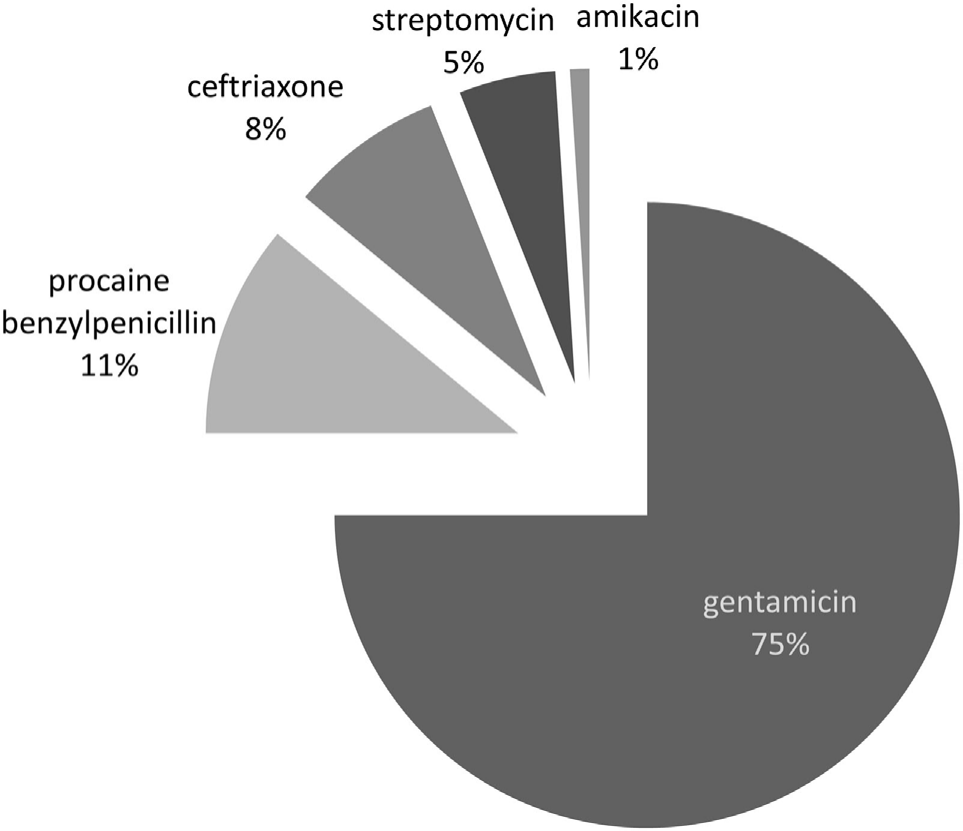
Parenteral outpatient antibiotic use in South Backa District expressed in percentage.

In SBD, 5.68% of total outpatient antibiotic consumption was used parenteraly. The three most commonly used antibiotic groups for parenteral treatment were the aminoglycosides (J01G; 81.04%), the penicillins (J01C; 11.11%) and the cephalosporins (J01D; 7.84%). The most commonly used parenteral antibiotics were gentamicin (75%), procain-benzylpenicillin (11%) and ceftriaxone (8%) (Figure 1). In Finland, only tobramycin in negligible percentage (0.05%) was used for parenteral treatment, while in Denmark no injectable drug appeared.

DU90% segment in SBD included 13 antibiotics (Table 8). Financial expenses for DU90% segment accounted for 91.3% of overall cost in J01 group, whereas 10% utilization in DDD accounted for 8.7% of overall cost.

**Table 8:** Antibiotics for systemic use (J01) within DU90% segment expressed in the number of DDDs/TID, and the cost per defined daily dose (DDD) in Euros within and beyond DU90% segment in south backa district (SBD).

No	ATC	INN	%	Defined daily doses per 1,000 inhabitants per day	Cost (euro)/DDD
1	J01CA04	Amoxicillin^a^	21.17	5.71	0.21
2	J01DB01	Cefalexin^a^	13.24	3.57	0.70
3	J01AA02	Doxycycline^a^	11.47	3.09	0.12
4	J01EE01	Co-trimoxazole^a^	6.91	1.86	0.24
5	J01FA10	Azythromycin^a^	6.38	1.72	1.52
6	J01MA02	Ciprofloxacin^a^	5.17	1.39	1.52
7	J01CE02	Phenoxymethylpenicillin^a^	4.98	1.34	0.65
8	J01GB03	Gentamicin	4.27	1.15	1.15
9	J01CR02	Amoxicillin+clavulanic acid^a^	4.25	1.15	0.83
10	J01CA01	Ampicillin	4.15	1.12	0.31
11	J01DD08	Cefixime^a^	4.01	1.08	2.16
12	J01FA09	Clarithromycin^a^	2.89	0.78	0.86
13	J01MB04	Pipemidic acid^a^	2.14	0.58	2.11
DU90% 1–13 (average)			91.04	24.54	0.95
Others 14–37 (average)			8.96	2.40	1.89

TOTAL 1–37			100.00	26.94	1.56

*ATC, anatomical therapeutic chemical; INN, International Nonproprietary Name*.

*^a^Drugs fully reimbursed by the Republic Fund for Health Insurance of Serbia in 2008*.

## Discussion

According to our knowledge, this study was first to examine the outpatient consumption of 100% sample of antibiotic (issued by prescription and bought without prescription) in one region in Serbia covering more than 6000,000 inhabitants; calculation of DU90% segment to estimate the prevalence and the structure of antibiotics, and the share of parenteral preparations in the total outpatient use.

We compared the results obtained with the drug utilization data in Denmark and Finland. These Scandinavian countries were chosen because they are stable middle-ranking countries according to the antibiotic consumption in Europe (2, 11). Aforementioned countries were also selected for comparison because of their well-developed pharmacotherapeutic practice. Namely, they possess publicly available data sets for drug consumption, well-established methods for drug consumption monitoring, as well as sufficient financial resources for constant development and implementation of pharmacotherapeutic treatment guidelines thereby providing their population with optimal treatment options.

The total outpatient utilization of antibiotics in SBD is high (26.93 DDDs/TID), although it is lower than the total use of antibiotics in Serbia according to the data retrieved from the annual report issued by the Agency for Drugs and Medical Devices of the Republic of Serbia for the same year (47.39 DDDs/TID) (6). This difference could be the consequence of the fact that our data were based on antibiotics issued on prescription in all state-owned and antibiotics bought without prescription in all private pharmacies in SBD, while national data was based on antimicrobial wholesale data. The total outpatient utilization of antibiotics in SBD is 1.6 times higher than in Denmark (9) and 1.48 times greater than in Finland (10), and it is in accordance with the utilization in Belgium (27.66 DDDs/TID), France (27.99 DDDs/TID), and Italy (28.45 DDDs/TID) (2), European countires with the highest outpatient antibiotic use. This is in accordance with the finding related to the first valid, representative, and comparable published data on antimicrobial use in Serbia, according to which Serbia is among the countries with an above average antibiotic use (12). Interestingly, high antibiotic consumption in our study reflects similar prescribing habits with surrounding countries. For example, in Montenegro (which was part of Yugoslavia and afterward the State Union of Serbia and Montenegro until 2006), the total outpatient amount of antibiotic was 39.29 DDDs/TID in 2009 (13). Furthermore, in Zagreb, the capital of Croatia, the total outpatient consumption was 38.31 DDDs/TID in 2007 (14). The similarities between these countries could be influenced by the common cultural, educational, and prescription behavior habits (15, 16).

Furthermore, the possibility to procure antibiotics without prescription could be one of the factors driving high consumption of antibiotics in SBD. Namely, out of the total antibiotic consumption in our study, almost 30% was bought in private pharmacies, most often without prescription. This study was conducted before the implementation of stricter laws on antibiotics purchasing without medical prescription in 2011 (17). Although antibiotics are not available without prescription nowadays, some private pharmacies in Serbia do not adhere to this regulation. Namely, a recent study conducted in Novi Sad as a major city of the SBD reported, 50% self-medicating with antibiotics during their lifetime and that 25% of the patients opted for self-medication during the last infection. These results indicated that self-medication rate is higher than in other countries in Europe (18). The self-medication practice with antibiotics with antibiotics in Europe from 3% in northern region to 30% in eastern Europe which is in accordance with the percentage of antibiotics bought without prescription recorded in our study (18, 19).

Although the consumption of penicillin drugs is prevailing in all three observed settings, there are some differences between the structure of their use. Unlike Denmark, in SBD and Finland, a higher use of broadspectrum penicillins was noticed, which is similar to other settings in Europe (20). However, the latest study related to antibiotic consumption in the primary care sector in Denmark also showed the increase in the use of broad-spectrum penicillins between 2004 and 2013, which is worrisome (21). Decreasing trend of ampicillin use has been in Europe in SBD ampicillin is still among 10 most frequently used antibiotics. The reason of this high consumpion could be the historical consumption habits of inhabitants; it has been the most popular antibiotic in Serbia for years (4, 22). The utilization of combination of penicillin with β-lactamase inhibitor in SBD (1.15 DDD/TID) was similar to the consumption in Finland but it was lower than in neighboring ex-Yugoslavia countries. In Croatia, the amoxicillin+clavulanic acid was with 5.34 DDD/TID leading drug in outpatients consumption (23), while in Montenegro (13) in 2009 year the total outpatient utilization of this antibiotic was 3.9 times greater than in SBD. It is interesting that in neighboring Hungary, amoxicillin+clavulanic acid was one of the most widely sold antibiotic without prescription (24).

Unlike Denmark, the only country in Europe that does not use cephalosporins in outpatients, in SBD cephalosporins represent almost 20% of total use of antibiotics (21). The first-generation cephalosporins represents more than 70% of the total cephalosporins use (mainly cephalexine) what is less than in Finland (95%), similar to the utilization found in Luxembourg, Israel and Croatia in 2008 (2). While the second-generation cephalosporins was used in less than 7% in SBD, the consumption of the third generation was high (more than 24%), comparable to a few countries (Italy, France) with the highest consumption of this generation of cephalosporins (25). The consumption of the second generation was mainly presented with cefaclor, although the dosage regime is inconvenient, whereas in Finland cefuroxime was the most frequently used cephalosporin of the second generation, a drug with better pharmacokinetc properties regarding dosing frequency. The reason of this high consumpion of cefaclor could be the historical consumption habits of inhabitants; it has been one of the most popular antibiotic in Serbia for years (5, 22). In addition, the third generation of cephalosporins are expensive drugs with a very broad antibacterial spectrum, that is why their irrational use contributes not only to the development of antibacterial resistance but also represents a significant impose significant financial burden on health expenses (26–28). In addition, this innapropriate use of the third generation for parenteral use presented by ceftriaxone in our study could be explained by its good pharmacokinetic properties such as once-daily administration, which is convinient for patients. Likewise, cefixime is an attractive option for outpatients for oral therapy, because of the broad antibacterial spectrum and once-daily dosing regime.

The consumption of co-trimoxazole in SBD (1.86 DDDs/TID) was higher than consumption of sulfonamides and trimetoprim in Finland, country with the highest consumption among 31 European countries participating in the ESAC project in 2008 (2). Despite the high resistance of *E. coli* isolated from the urine of outpatients in SBD to co-trimoxazole (36.23%) in 2008 as well as in 2012, it is still first- choice agent for the treatment of uncomplicated urinary infections outpatients according to the national guideline for antimicrobial drug use (issued in 2004) in Serbia (29, 30). Because co-trimoxazole is financialy affordable (0.24euro/DDD), it has been commonly used for various infections in Serbia. Increased resistance to these drugs is a problem not only in outpatients but also particularly in inpatients in Serbia (29–31). Resistence to co-trimoxazole among isolates of E. coli ranges from 10 to 70% in different part of the world (32). Therefore, co-trimoxazole may no longer be effective in the treatment of *E. coli* strains resistant to this antibiotic. This should be taken into consideration in the making and updating of pharmacotherapeutic guidelines in Serbia.

Doxycyclin was among the three most frequntly used antibiotics in outpatients in our study, despite the limited number of indications for its administration nowadays. The high consumption was a consequence of several reason: low price (the cheapest antibiotic with the price of 0.12euro/DDD), possibility to buy it without prescription in private pharmacies before the restriction of free sale of antibiotics in 2011 and convenient administration once a day. As for the total consumption of tetracycline in Serbia, a gradual decrease in the utilization was recorded from 2006 (4.58 DDD/TID) to 2015 (2.25 DDD/TID) (33, 34). However, according to the recent survey aimed to report the first valid, representative, and comparable data on antimicrobial use in non-European Union countries of the WHO European region, Serbia is still among the countries with the highest use of tetracycline (12).

Similarly, high use of macrolide was noted in SBD, mainly azithromycin, whereas the use in Denmark and Finland was lower 2.4 and 1.27 DDD/TID, respectively. According to the above mentioned study on antimicrobial use in the non-European Union southern and eastern European countries, Montenegro and Serbia were the highest consumers of macrolides, mainly azithromycin. According to the guides for good clinical practice, issued by Ministry of Health of the Republic of Serbia, penicillins and/or macrolides are recommended as first line therapy for the treatment of respiratory infections in adults, which are the most common infections in outpatients (35).

Besides this, the once a day regimen and good safety profile of azitromycin contributes to the frequent us of macrolide antibiotics for empirical therapy in Serbia.

In relation to the group of macrolides, an increase in the overall consumption of these medications is also noticable in the whole of the Serbia from 2006 (3.55 DDDs/TID) to 2015 (5.34 DDDs/TID).

The utilization of quinolones was several times higher than in Scandinavian countries, with ciprofloxacine being the most widely used fluorinated quinolone. Use of ciprofloxacine was 2.5 and 4.3 times higher than in Finland and Denmark. The first- and the second generations of quinolones were most commonly used in SBD, which is comparable to the utilzation in most countries in Europe (2). The utilization of pipemidic acid (0.58 DDDs/TID) was higher than in Italy (0.25 DDDs/TID), where the consumption of pipemidic acid is the highest within ESAC participating countries (36). Quinolones are not recommended as a drug of first choice for the treatment of many infectious diseases, their high use recorded in our District raises concern regarding their appropriate use, especially for the treatment of multi-drug-resistant infections, such as tuberculosis.

The proportion of outpatient parenteral antibiotic use in SBD (5.68%) was in accordance with the proportion in 20 European countries in 2006, where it ranged from 0.001% in Iceland to 6.75% in Russia (37). The tree most commonly used groups were the same in European countries as in SBD, but with different order and proportion: cephalosporins (44.58%), the aminoglycosides (25.27%) and the penicillins (17.78%). The high utilization of aminoglycosides in SBD represented almost completely with gentamicin is a result of empirical prescribing of gentamicin by the general practitioner without the previous antibiogram usually for the treatment of urinary tract infections in outpatients (29).

In SBD and Finland, the prescription of antibiotics was split among greater number of compounds (37 and 31, respectively) than in Denmark (only 19 antibiotics). An interesting fact is that DU90% segment included 13 drugs in all three compared countries. In SBD, the cost/DDD within DU90% segment was 0.95 EUR, whereas the cost/DDD beyond this segment was 1.89 EUR/DDD, indicating that the cheaper antibiotics were more often used than the expensive ones (38, 39). In Finland these figures were 1.08 EUR/DDD and 3.46 EUR/DDD and in Denmark 3.1 EUR/DDD and 0.88 EUR/DDD, demonstrating that cheaper drugs were consumed in SBD and Finland than in Denmark (8, 9).

One of the reasons for the difference in average price within DU90% segment is the high price of phenoxymethylpenicillin in Denmark (9.74 EUR/DDD), the most used antibiotic, representing almost 33% of the prescription. In Denmark, only six antibiotics were beyond DU90% segment with the most expensive drug within this segment being sulfamethizol with 1.54 EUR/DDD (representing only 1.85% of the prescription).

The limitation of the study was the lack of follow up the patients indications. As with any study on drug consumption, it was not possible to compare the objective compliance use with the dosing regime. The study was conducted in SBD, Serbia which may not be representative for Serbia as a whole, due to distinct socieconomic and cultural characteristics.

### Strength of the Study

The strength of this study is 100% sample of antibiotics used from one area in Serbia, covering more than 600,000 inhabitants. So far such data were not available in Serbia for the follow up and the comparison of use of antibacterials. Also, extensive application of ATC/DDD classification system, and the drug utilization 90% method to assess the prevalence and the structure were shown in order to analyze the structure of antibiotics.

## Conclusion

Our study on the utilization of antibiotics in SBD, which accounts almost 8.2% of the Serbian population, indicate high utilization in Serbia as a whole. The comparison of our data with those from Scandinavian countries for the year 2008, the use of antibacterials is significantly higher in SBD. However, the use of antibiotics is still not as high as in countries with the highest outpatient consumption in Europe.

Irrational use of ampicillin, III generation cefalosporins, co-trimoxazole, and gentamicin, as showed in our study, will aggravate the existing problem of antimicrobial resistance, leading to further increase in the morbidity and mortality of infections caused by resistant bacteria and treatment-related costs due to the lack of an appropriate treatment.

Interventions to improve antibiotic use and education on rational antibiotic use should be essential for this District. Besides national monitoring of antibiotic consumption, availability of internationally comparable data on antibacterial consumption would be a valuable oportunity for continuous comparison of our consumption with those in other countries in Europe. Furthermore, differences in antibiotic consumption between SBD and Scandinavian countries, indicate the need of updated guidelines for in- and especially outpatients regarding rational antimicrobial drug use in Serbia.

## Author Contributions

All the authors have provided substantial contributions to the development of the manuscript. OH, AS, and ZT contributed to the overall conception and design. VM and BM gathered the data. MK, AT, OH, and AS analyzed the data. All the authors contributed to the interpretation of the data and the drafting of the manuscript. All the authors have given final approval for the paper to be published in Frontiers and agree to be accountable for the content presented therein.

## Conflict of Interest Statement

The authors declare that the research was conducted in the absence of any commercial or financial relationships that could be construed as a potential conflict of interest.

## Abbreviations

ATC, anatomical therapeutic chemical; DDD, defined daily dose; DDDs/TID, defined daily doses per 1,000 inhabitants per day; DU90%, drug utilization 90% segment; ESAC, European Surveillance of Antimicrobial Consumption; SBD, south backa district.
